# Supplementary material for: Binding and Neutralizing Capacity of Respiratory Syncytial Virus (RSV)-Specific Recombinant IgG Against RSV in Human Milk, Gastric and Intestinal Fluids from Infants
Source: Nutrients. 2020 Jun 27;12(7):1904. doi: 10.3390/nu12071904 (PMC7400675; doi:10.3390/nu12071904)
Supplement: Supplementary file 1 [file nutrients-12-01904-s001.pdf]

Supplemental Materials

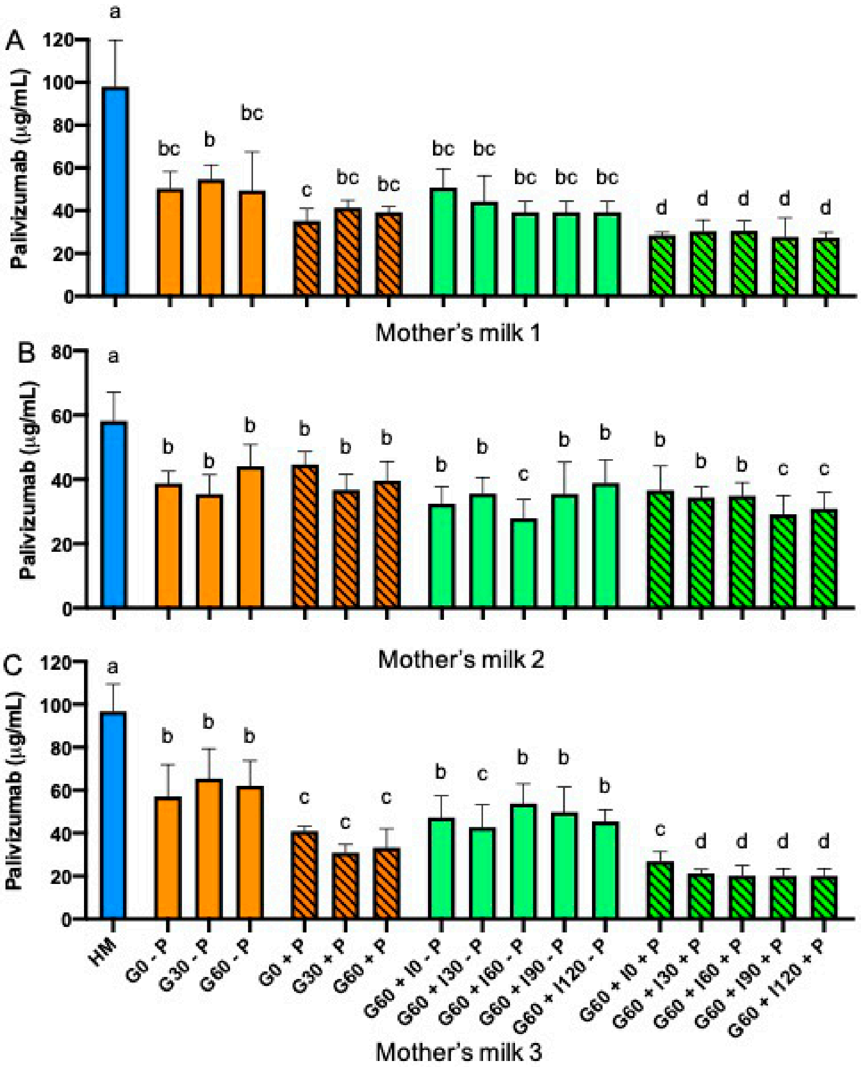

Figure S1A–C. Binding capacity of palivizumab across simulated term infant digestion in three different mother's milk samples. (A–C) Palivizumab concentration in human milk (HM) during simulated infant digestion; in gastric conditions at 0 min (G0 – P), at 30 min (G30 - P) and at 60 min (G60 – P) without protease (pepsin); in intestinal conditions at 0 min (G60 + I0 – P), at 30 min (G60 + I30 – P), 60 min (G60 + I60 – P), 90 min (G60 + I90 – P) and 120 min (G60 + I120 – P) without protease (pancreatin). Palivizumab concentration in human milk (HM) during simulated infant digestion; in gastric conditions at 0 min (G0 + P), at 30 min (G30 + P) and at 60 min (G60 + P) with protease (pepsin); and in intestinal conditions at 0 min (G60 + I0 + P), at 30 min (G60 + I30 + P), 60 min (G60 + I60 + P), 90 min (G60 + I90 + P) and 120 min (G60 + I120 + P) with protease (pancreatin). Values are mean ± SD from 6 replicates for each condition. Letters a, b, c and d show statistically significant differences between groups ($P < 0.05$) using one-way ANOVA followed by Tukey's multiple comparison tests.

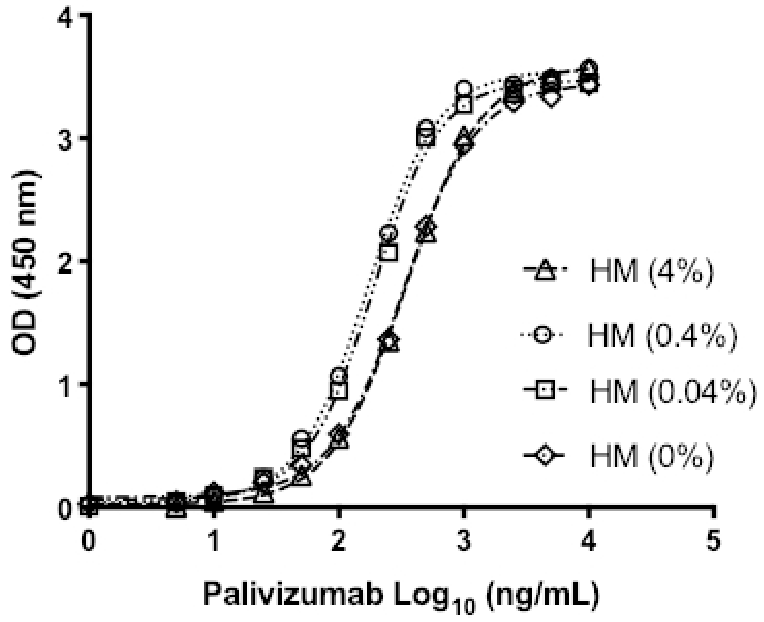

Figure S2. Standard curve with palivizumab detection in binding buffer (PBST + 10% human serum) containing 0–4% of mixed human milk.

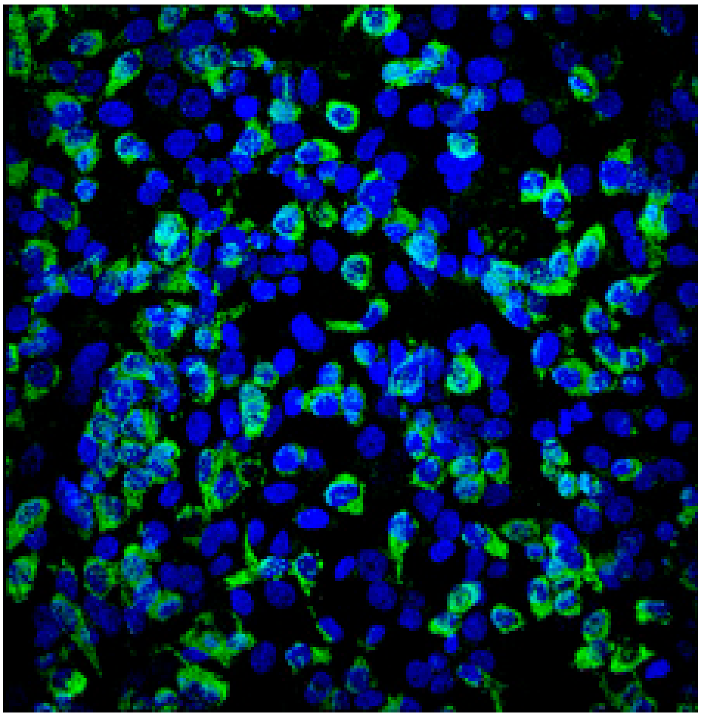

Figure S3. Hep-2 cells infected for day 3 incubated with RSV-GFP (50% tissue culture infectious dose: 3.4 x 104 focus forming units/mL) using the confocal microscope 3-D image (40x objective).
